# A versatile distance-based approach for gene expression selection across diverse biological systems

**DOI:** 10.3389/fimmu.2026.1843796

**Published:** 2026-07-13

**Authors:** Qiaoling Ye, Rodney Macedo, Laura Martinez-Verbo, Vytaute Plekaviciute, Jana Vazquez Navarro, Elisabet Garcia, Joan Pagès-Oliveras, Juan-José Lozano, Cecilia Cabrera, Aida Perramon-Malavez, Daniel López, Clara Prats, Maria-Rosa Sarrias

**Affiliations:** 1 Innate Immunity Group, Germans Trias i Pujol Research Institute (IGTP), Badalona, Spain; 2 Departament de Física, Institute for Research and Innovation in Health (IRIS), Universitat Politècnica de Catalunya – BarcelonaTech, Castelldefels, Spain; 3 Irsicaixa, Hospital Universitari Germans Trias i Pujol, Badalona, Spain; 4 Germans Trias i Pujol Research Institute (IGTP), Badalona, Spain; 5 Bioinformatics Platform, Biomedical Research Network on Hepatic and Digestive Diseases (CIBEREHD), Instituto de Salud Carlos III, Madrid, Spain; 6 Biomedical Research Network on Hepatic and Digestive Diseases (CIBEREHD), Instituto de Salud Carlos III, Madrid, Spain

**Keywords:** differential gene expression, macrophage polarization, mathematical algorithm, mRNA-seq analysis, polarization speed

## Abstract

**Introduction:**

Differential gene expression analysis is essential for characterizing immune cell phenotypes, yet conventional approaches—typically based on log_2_ fold−change (log_2_FC) and False Discovery Rate (FDR) thresholds—often struggle to capture the complexity and continuum of transcriptional states.

**Methods:**

To address this limitation, we developed a new computational method for gene selection from mRNA−seq data: the Cartesian Distance−Based Gene Expression (CDBGE) selector. This algorithm identifies differentially expressed genes by leveraging multidimensional expression distances rather than relying on traditional univariate statistical cutoffs, enabling a more refined and biologically coherent gene−marker selection.

**Results:**

We applied the CDBGE selector to construct a gene−based framework for distinguishing macrophage polarization states. The model was trained using publicly available macrophage transcriptomic datasets and subsequently validated with *in vitro* human macrophages stimulated with IFN−γ/LPS, conditioned medium from HepG2 liver cancer cells (Sec-HepG2), or IL10. To evaluate its generalizability beyond macrophage biology, we further tested the method on human embryonic stem cell differentiation datasets. Compared with standard differential expression pipelines, the CDBGE selector more effectively identified subtype−specific markers and revealed dynamic transcriptional transitions over time.

**Discussion:**

These findings demonstrate that distance−based gene selection provides an improved strategy for analyzing complex mRNA−seq datasets. Overall, the CDBGE selector offers a robust, scalable, and broadly applicable tool for differential gene expression analysis and phenotype characterization.

## 1 Introduction

Cellular phenotypes are highly heterogeneous and context-dependent. Mathematical modeling to explore cellular dynamics offers an interesting approach to fine tune this heterogeneity (1). These models rely on mathematical equations and parameterized cell behaviors to capture cell-cell interactions and cell-environment interaction (2, 3). However, these classifications rely on simplified, deterministic factors. Consequently, traditional mathematical models are limited in their capacity to incorporate the full complexity of cellular behavior. More recently, computational approaches, particularly image-labeling-based machine learning (ML), have shown promise in integrating large and diverse datasets, and have been increasingly applied to cellular labeling. Compared with earlier bioinformatics methods, whose accuracy in classifying cellular populations is often limited, this approach provides a rapid and cost-effective means to accelerate the discovery of cellular phenotypic diversity (4–6). Yet, it is limited in its capacity to reveal the genetic and molecular mechanisms underlying these phenotypic variations. This gap underscores the growing need for approaches that utilize genetic and transcriptomic information, enabling a more comprehensive understanding of cellular diversity and function.

Macrophages (a type of immune cell) are a paradigmatic example of the need to increase our ability to classify cellular diversity. These are highly dynamic cells that play key roles in development, homeostasis, tissue repair, and immune response (7). To exert their roles, these cells exert trophic, regulatory, repair, and effector functions (7). Macrophages originate from tissue-resident precursors or circulating monocytes, and they adapt these functions according to local environmental signals (7, 8). In this regard, a hallmark of macrophages is their remarkable plasticity, i.e. their ability to adopt distinct phenotypes and activate different gene expression and functional programs in response to microenvironmental cues (9). Referred to as polarization, in this adaptive process, classical M1 and alternative M2 activation states represent two extremes of a dynamic spectrum of macrophage activation. M1-type macrophages are pro-inflammatory and highly phagocytic, whereas M2-type macrophages are immunosupressive and support tissue repair (9). However, this traditional binary M1/M2 polarization classification is increasingly recognized as inadequate. Under diverse stimuli, macrophages show complex and versatile phenotypes that do not fit neatly into this dichotomy (10).

A clear example of this heterogeneity arises from studies on cancer. Macrophages are a major component of the tumor microenvironment (TME), often accounting for 50% of tumor-infiltrating cells (11). Within the TME, they are commonly referred to as tumor-associated macrophages (TAMs), a population strongly linked to immunosuppression and tumor progression (11). Being highly plastic, TAMs can transition between states in response to tumor-specific signals, leading to transcriptional rewiring and the emergence of distinct gene expression profiles (7). These observations highlight the need to redefine TAM subtypes to better understand their functional diversity and their association with patient prognosis.

In this study, we developed a straightforward mathematical algorithm to select genes that enable macrophage subtype classification under each experimental condition. Our approach prioritized interpretability, versatility, simplicity, and flexibility while maintaining accuracy across datasets from different species and experimental conditions. To demonstrate the robustness and generalizability of this method, we applied it to both human and murine macrophage datasets, which included samples treated with various stimuli to induce different phenotypes, including TAM-like phenotypes. We further extended the method to analyze macrophage polarization dynamics over time, revealing the time at which macrophages exposed to a given stimulus diverge into phenotypes distinct from those induced by alternative stimuli. We further demonstrate that the algorithm enhances conventional gene selection strategies beyond macrophages, applying it to stem cell differentiation studies thereby providing a solid framework for differential gene expression (DGE) analysis.

## 2 Materials and methods

### 2.1 Primary cells and cell lines

All studies involving human samples were conducted following the principles of the Declaration of Helsinki and current legislation on the confidentiality of personal data and were approved by the Human Ethics Committee of the Hospital Universitari Germans Trias i Pujol (Code PI-24-299). Buffy coats, provided by the Blood and Tissue Bank (Barcelona, Spain), were obtained from healthy blood donors following the institutional standard operating procedures for blood donation and processing, including informed consent (CEIM Vall d’Hebron’ Hospital, Barcelona, Code 250007). Peripheral blood mononuclear cells (PBMCs) were isolated by Lymphoprep (515967, Serumwerk Bernburg) density-gradient centrifugation at 720 × *g* for 30 min. Recovered cells were washed twice in phosphate-buffered saline (PBS) and counted using NucleoCounter NC-3000, following the manufacturer’s instructions. Peripheral blood monocytes (PB monocytes) were isolated by adherence in a 5% CO_2_ incubator at 37 °C in RPMI-1640 with L-glutamine (10-040-CV, Corning) supplemented with 10% heat-inactivated human AB serum (H4522, Sigma–Aldrich) for 30 min. Non-adherent cells were removed, while adherent cells were washed twice with PBS (12899712, Fisher). The purified cells were cultured in RPMI-1640 with L-glutamine, 10% heat-inactivated fetal bovine serum (FBS) (DE14-840E, Basel, Switzerland), 100 U/ml penicillin and 100 µg/ml streptomycin (P/S; P0781, Sigma–Aldrich), as previously described (12). To achieve mature macrophages, 50 ng/mL human recombinant macrophage colony-stimulating factor (M-CSF) (300-25, Peprotech) was added to the culture for 5 days.

HepG2 (RRID: CVCL_0027) cells were purchased from the ATCC (The American Type Culture Collection; Manassas, VA, USA) and cultured in EMEM supplemented with 2 mM glutamine (Lonza), P/S, and 10% heat-inactivated FBS. All human cell lines were identified and validated using the AmpFLSTR™ Identifiler™ Plus PCR Amplification Kit (A26364, Thermo Fisher Scientific) and the GeneMapper v3.2 software (RRID: SCR_014290). All cell lines tested negative for Mycoplasma. HepG2 conditioned media (Sec-HepG2) was obtained by growing cells to 90% confluency. The cells were then washed with PBS, and the medium was replaced with one containing 2% FBS. 24 h later, the supernatant was collected and centrifuged at 10000 rpm for 10 min at 4 °C to remove cellular debris. It was then aliquoted and stored at −80 °C for subsequent experiments, in which it was diluted 1:2 with media containing 10% FBS, described before (12).

### 2.2 *In vitro* polarization of macrophages

Mature macrophages (5 × 10^5^ cells/well) were incubated in 50 ng/ml M-CSF culture medium (control), or 50 ng/mL IFN-γ (Peprotech, 300-02-A, Rocky Hill, NJ, USA) plus 100 ng/mL LPS from *Escherichia coli* O111:B4 (14391, Sigma–Aldrich) (IFN-γ_LPS), 50 ng/mL IL10 (200-10-A, Peprotech), or Sec-HepG2 in 50 ng/mL M-CSF (12). Three biological replicates were collected for mRNA-seq analysis at 6 h, 24 h, and 48 h.

### 2.3 RNA extraction and gene expression profiling analysis

Macrophages were washed with PBS and disrupted with QIAzol Lysis Reagent (217084, Qiagen), and RNA was extracted using the miRNeasy Mini Kit (217084, Qiagen). mRNA-seq with three biological replicates was performed by the Genomics Platform at the Centre for Genomic Regulation (CRG, Barcelona). RNA integrity was assessed using a Fragment Analyzer with the Standard Sensitivity RNA kit (DNF-471, Agilent). Libraries were prepared from total RNA using the TruSeq Stranded mRNA Library Prep Kit (20020595, Illumina) and following the manufacturer’s instructions, generating strand-specific cDNA libraries suitable for cluster generation and sequencing on the NovaSeq 6000 platform using a 2×50 bp paired-end strategy with 30 million reads per sample and a 100-cycle S2 flow cell.

Skewer v0.2.2 (13) was used to remove the low-quality reads and trimming the Illumina adapter. STAR program (14) against genome (GRCh38) was used for mapping the reads followed by the quantification of genes with the RSEM (RNA-Seq by Expectation-Maximization) software package (15) for estimating gene and isoform expression levels from RNA-Seq data, using GENCODE v26 reference annotation (16). Genes with RSEM expected counts ≤ 5 across all samples were removed prior to differential expression analysis. DESeq2 (17) was carried out, a method for differential analysis of count data, using shrinkage estimation for dispersions and fold changes to improve stability.

### 2.4 Gene set enrichment analysis

GSEA was performed using the *clusterProfiler* R package (v4.16.0) (18). Differentially expressed genes were ranked by log_2_ fold change (log_2_FC), and gene identifiers were assigned based on HGNC symbols. The analysis was conducted with the *GSEA* function (19) using the KEGG gene sets obtained from the MSigDB collection (c2.cp.kegg.v2023.1.Hs.symbols.gmt). Parameters were set to a minimum gene set size of 1, a maximum gene set size of 100,000, and an epsilon of 1e−10. P-values were adjusted using the *Benjamini–Hochberg* method (20). Enrichment results were extracted from the GSEA output and used to identify significantly enriched KEGG pathways.

### 2.5 Algorithm development for the selection of genes that best discriminate between differently polarized macrophages

The genes used to distinguish macrophages were selected based on a Cartesian distance calculation with regard to the gene expression in a control group, giving rise to what we call the new Cartesian Distance-Based Gene Expression (CDBGE) selector. This method is based on the one developed in previous work (21). Before this process, and to reduce the complexity of the analysis, a pre-selection step was performed, in which only genes with an FDR< 0.05 were included.

An initial normalization process was applied independently to all gene expression measurements at each time point. Let us denote *Max_g_*,*_t_*,*_d_* as the maximum genetic expression of each gene (*g*) comparing the control (M-CSF) at the initial time with IFN-γ_LPS (IFNγ_LPS), IL10 and Sec-HepG2 (HepG2) values at a given timepoint (*t*), each donor (*d*) is calculated independently (Equation 1):

(1) $$Max_{g,t,d}=\max\left\{Control_{g,initial\;time,d},IFN\text{γ}\_\text{LP}S_{ɡ,t,d},IL10_{g,t,d},HepG2_{g,t,d}\right\}$$

We then normalized all gene expressions by dividing their values by the corresponding *Max_g_*,*_t_*,*_d_* at each time point, obtaining the normalized genetic expression of different macrophage types, *Normalized_g_*,*_t_*,*_d_*, at each time point (Equation 2).

(2) $$Normalized_{g,t,d}=norm\left\{\frac{Control_{g,\;initial\;time,d\;}}{Max_{g,t,d}},\frac{IFN{\text{γ}}_{\_}{\text{LPS}}_{\text{g,t,d}}}{Max_{g,t,d}},\frac{IL10_{g,t,d}}{Max_{g,t,d}},\frac{HepG2_{g,t,d}}{Max_{g,t,d}}\right\}$$

The distances of the IFNγ_LPS-, IL10-, and HepG2- stimulated groups were measured at each time step with respect to the control group (M-CSF) at the initial time. This calculation was performed for each donor (*d*) (Equations 3–5).

(3) $$dist\;IFN\text{γ}\_LPS_{g,t,d}=(Normalized_{g,t,d}\;IFN\text{γ}\_LPS-Normalized_{g,initial\;time,d}\;Control)$$

(4) $$dist\;IL10_{g,t,d}=(Normalized_{g,t,d}\;IL10-Normalized_{g,initial\;time,d}\;Control)$$

(5) $$dist\;HepG2_{g,t,d}=(Normalized_{g,t,d}\;HepG2-Normalized_{g,initial\;time,d}\;Control)$$

To assess the global distance of each macrophage group relative to the control group across all time points, we applied the following equation. This calculation was performed individually for each donor (*d*) and gene (*g*) (Equations 6–8).

(6) $$dist_{g,all_{time},d}IFN\text{γ}\_LPS=\sqrt{{\left(\overset{N}{\underset{t=1}{\sum }}dist\;IFN\text{γ}\_LPS_{g,t,d}\right)}^{2}}$$

(7) $$dist_{g,all_{time},d}IL10=\sqrt{{\left(\overset{N}{\underset{t=1}{\sum }}dist\;IL10_{g,t,d}\right)}^{2}}$$

(8) $$dist_{g,all_{time},d}HepG2=\sqrt{{\left(\overset{N}{\underset{t=1}{\sum }}dist\;HepG2_{g,t,d}\right)}^{2}}$$

Once the global distance between each macrophage group and the control group had been calculated for each donor and gene, we averaged the global mean distance for genes (*g*) of each macrophage type across all donors (*d*) (Equations 9–11).

(9) $$mean_{dist,g}\;IFN\text{γ}\_LPS=mean\left\{dist_{g,all_{time},d}\;IFN\text{γ}\_LPS\right\}$$

(10) $$mean_{dist,g}IL10=mean\left\{dist_{g,all_{time},d}IL10\right\}$$

(11) $$mean_{dist,g}HepG2=mean\left\{dist_{g,all_{time},d}HepG2\right\}$$

We then computed the difference between the global mean distance of each gene to selected macrophage markers, in the following way. For IFNγ_LPS macrophages, we calculated the difference between (*mean_dist,g_* IFNγ_LPS – *mean_dist,g_* IL10), selecting those that provided a positive distance as potential markers and ranking them from highest to lowest positive values. Similarly, we selected as potential IL10 markers the genes that give rise to a positive value when computing the difference between *mean_dist,g_* IL10 and *mean_dist,g_* IFNγ_LPS, also ranking them from highest to lowest distances values. Finally, the same approach was applied for the selection of potential markers for HepG2, based on the difference between *mean_dist,g_* HepG2 and *mean_dist,g_* IFNγ_LPS and ranking them according to the result, as well. The final choice of the three gene sets representing the three profiles was done by fixing a number of genes, *n_g_*, and selecting those with highest values in the previous rankings.

### 2.6 Analysis of polarization speed

The rate of polarization was calculated as follows, based on the principle that velocity can be expressed as the ratio of distance to time. It was computed in a multidimensional spatial framework that accounts for changes in time along distance of all gene clusters (i.e., velocity towards each of the 3 profiles), followed by the calculation of the modulus. Therefore, speed is the quadratic sum of the difference in distance along each gene cluster divided by the given time interval. This calculation was performed for each donor (*d*), but considering only the cluster of *n_g_* genes selected in each case (Equation 12).

(12) $$Speed_{t,d}=\frac{1}{\left(t_{i+1}-t_{i}\right)}\sqrt{{\left(dist_{cluster}\;IFN\text{γ}\_LPS_{ti+1,d}-dist_{cluster}\;IFN\text{γ}\_LPS_{ti,d}\right)}^{2}+{\left(dist_{cluster}IL10_{ti+1,d}-dist_{cluster}IL10_{ti,d}\right)}^{2}+{\left(dist_{cluster}HepG2_{ti+1,d}-dist_{cluster}HepG2_{ti,d}\right)}^{2}}$$

where *t* is time, $dist_{cluster}\;IFN\text{γ}\_LPS\;$ is the distance of the genetic expression of the selected IFN-γ_LPS gene set, $dist_{cluster}IL10$ is the distance of the genetic expression of the IL10 gene set, and $dist_{cluster}HepG2$ is the distance of the genetic expression of the HepG2 gene set.

### 2.7 Established methods for gene selection in comparative analysis

Gene expression, measured as log_2_FC relative to M-CSF, together with statistical significance assessed using the false discovery rate (FDR), was used as selection criteria (conventional method) (17). For each macrophage group, genes were first pre-selected by applying thresholds of log_2_FC > 1 and FDR< 0.05 across all time points. The mean log_2_FC was then computed for the remaining genes, and group-specific markers were defined as those with the *n_g_* highest mean log_2_FC values.

Random Forest method was implemented using *random forest* package in R (22). The model was trained on gene expression data, with the number of trees set to 500 to ensure stability of the estimates. Variable importance was assessed using the mean decrease in Gini index. All genes were ranked according to their importance scores, and the top 27 genes with the highest importance values were selected.

A weighted gene co-expression network analysis (WGCNA) was performed using the *WGCNA* package in R (23). Gene modules were identified based on co-expression patterns, and their relationships with the phenotype were evaluated using module–trait correlations. The module showing the strongest association with the trait and a statistically significant correlation with the studied phenotype was selected.

### 2.8 Evaluation of the gene selection methods and statistical analyses

To evaluate whether the selected genes can accurately define the phenotype of each group, clustering analysis was performed using the *mclust* package in R (24). Samples were assigned to groups based on expression profiles of the selected gene set. Clustering results were visualized by the heatmap generated with the *pheatmap* R package (25). Performance was evaluated by constructing a confusion matrix to quantify concordance between predicted group assignments and known labels. In parallel, principal component analysis (PCA) was conducted using the *factoextra* package in R (26) to examine the extent of separation between groups in reduced-dimensional space.

Resulting p-values were automatically adjusted for multiple comparisons using the Benjamini–Hochberg method, and p-values less than 0.05 were considered statistically significant. Graphs were generated using the *ggplot2* (v3.5.2) and *ggpubr* (v0.6.0) R packages (27, 28).

### 2.9 Databases for modeling development

To develop the model, we used two publicly available gene expression datasets. The first dataset (GSE158094) contains RNA-seq data from murine macrophages with three biological replicates per condition. Macrophages were polarized using 100 ng/mL LPS in combination with 25 ng/mL IFN-γ to induce an M1-like phenotype, or 25 ng/mL IL4 to induce an M2-like phenotype. A control condition with 20 ng/mL M-CSF was also included. Samples were collected at multiple time points, namely 1, 2, 4, 6, 12, 24, and 72 h post-stimulation, thereby capturing a wide temporal transcriptional response (29).

In addition, we included data from the GSE16385 dataset, which provides microarray-based gene expression profiles of human macrophages. This dataset contains one replicate per condition, with macrophages polarized using 100 ng/mL IFN-γ and 50 ng/mL tumor necrosis factor (TNF), or 100 ng/mL IL4, alongside a control condition treated with 20 ng/mL M-CSF. Samples were collected at 4, 12, 24, and 72 h after stimulation (30).

### 2.10 Model validation

To validate the applicability of the CDBGE selector in different databases and cell types, we applied the algorithm to the GSE273627 and GSE274620 datasets. The former included RNA-seq data of human macrophages polarized using either 10 pg/mL LPS combined with 20 ng/mL IFN-γ or 20 ng/mL IL4, with three biological replicates per condition, and collected at 24, 48, and 72 h post-stimulation (31). The latter dataset included mRNA-seq data from an *in vitro* model of human embryonic stem cell (hESC) differentiation into cardiomyocytes via the mesodermal lineage, with two biological replicate and samples collected at 10 defined time points: 0, 1, 2, 3, 4, 6, 8, 10, 12, and 18 days (32).

## 3 Results

### 3.1 Generation of a novel gene expression selector based on Cartesian distances using public data

**Figure 1:**
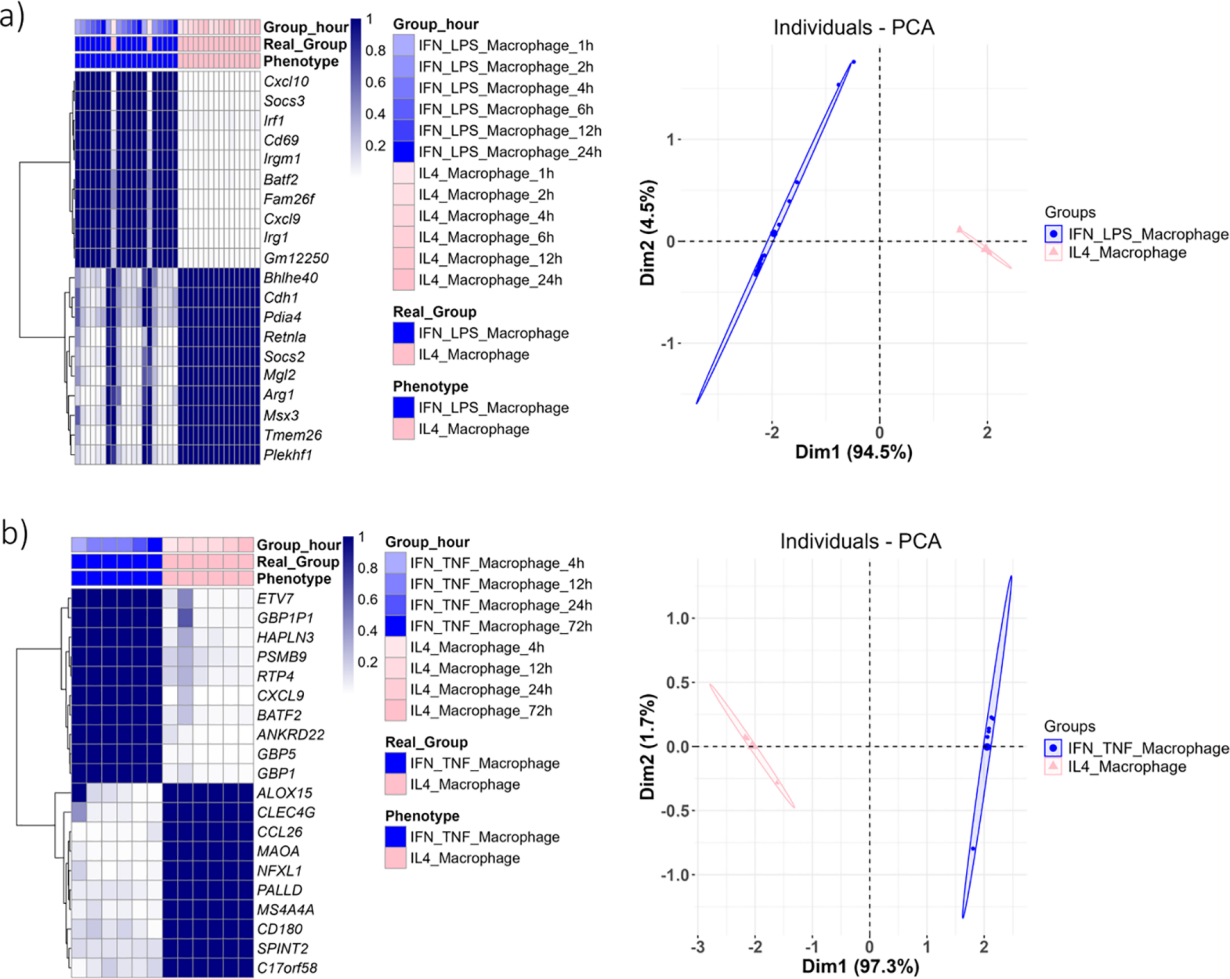
Determination of alternative macrophage gene markers by the new CDBGE selector. Publicly available data on *in vitro* polarized macrophages from: **(A)** the mouse macrophage GSE158094 database and **(B)** the human macrophage GSE16385 database were used to develop the CDBGE selector. Left: heatmap of 10 selected genes/condition selector. Right: PCA scatterplot of the expression profile of the 10 selected genes for each condition. Percentages represent variance captured by Dimension (Dim) 1 and Dim 2.

To develop the CDBGE selector, we used publicly available gene expression data from mouse (Liu et al., GSE158094) (29) and human (Szanto et al., GSE16385) (30) macrophages polarized *in vitro*. Feature selection identified a set of genes that enabled the model to correctly classify 94% and 100% of cell phenotypes, respectively, according to PCA and confusion matrix analyses (**Figure 1**, **Supplementary Table 1**). From GSE158094, genes *Cxcl10, Socs1, Irg1, Ccl12, Socs3, Cxcl9, Cd69, Gm12250, Irg1, and Irgm1* were selected in IFN-γ_LPS macrophages, while *Arg1, Retnla, Tmem26, Ccl24, Ccl7, Plekhf1, Socs2, Mgl2, Hbegf, and Pdcd1lg2* were selected in IL4 macrophages (**Figure 1A**). Two samples, both from time point 1 h were misclassified, suggesting potential overlap or transitional gene expression profiles at this early time.

Likewise, when applied to human macrophage data from GSE16385, the CDBGE selector identified *CXCL9, ANKRD22*, *HAPLN3*, *ETV7, BATF2, GBP5, GBP1, GBP1P1, PSMB9* and *RTP4* as upregulated genes indicative of the IFN-γ_TNF profile, and *CCL26, MAOA, NFXL1, PALLD, SPINT2, C17ORF58*, *CLEC4G, MS4A4A*, *ALOX15*, and *CD180* for the IL4 profile (**Figure 1B**). In this case, genes were pre-selected based on p-values< 0.05 obtained from moderated t-tests, since the comparison between IFN-γ + TNF–stimulated and M-CSF–derived macrophages did not yield a sufficient number of genes passing the FDR< 0.05.

In both studies, PCA and confusion matrix confirmed that the selected markers successfully distinguished between differently polarized macrophages, as each of the two groups formed distinct clusters (**Figure 1**, **Supplementary Table 1**).

### 3.2 Success of *in vitro* macrophage polarization *ad-hoc* experiment

**Figure 2:**
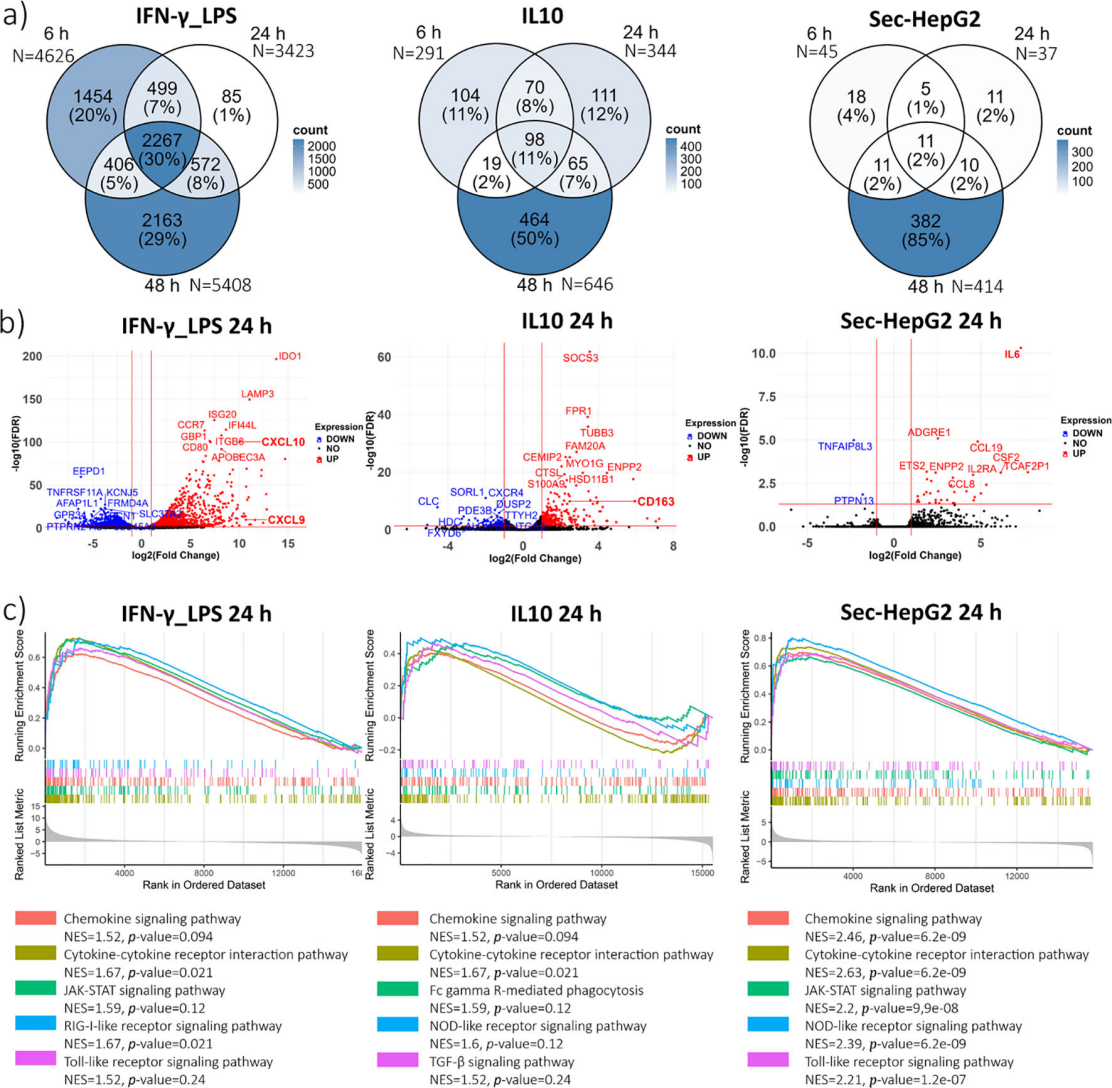
*In vitro* polarization of human macrophages over time reveals stimulus and time-specific differences in gene expression. **(A)** Venn diagram of differentially expressed genes (FDR<0.05, log2FC<-1 & >1) at 6 h, 24 h, and 48 h in IFN_LPS (left), IL10 (center), and Sec-HepG2 (right) vs. M-CSF control as detected by RNA-seq data sets. **(B)** Volcano-plot representing genes differentially expressed at 24 h in INF-γ_LPS (left), IL10 (center), and Sec-HepG2 (right) vs. M-CSF control. Red and blue dots mark the genes with significantly increased (right) or decreased (left) expression (p< 0.05), respectively. The red line is set to a p-value of 0.05. **(C)** GSEA plots of DGE for IFN-γ_LPS (left), IL10 (center), and Sec-HepG2 (right) vs M-CSF macrophages at 24 h.

We conducted differential gene expression (DGE) analysis on human peripheral blood monocyte-derived macrophages isolated from 3 healthy donors. For this, cells were treated with the standard polarization stimuli IFN-γ_LPS and IL10, as well as with conditioned medium of liver cancer cells (Sec-HepG2), for 6, 24, and 48 h, and their expression profiles were compared to unstimulated M-CSF matured macrophages (**Figure 2**).

IFN-γ_LPS macrophages showed significant changes in gene expression across multiple time points (**Figure 2A**). Considering a log_2_FC > 1 and<-1, FDR< 0.05, at 6 h post-treatment, 4,626 genes were significantly differentially expressed vs. M-CSF matured macrophages, with 1,454 of these being unique to this time point. At 24 h, 3,423 genes showed significantly increased expression, with only 85 unique to this time. By 48 h, the number of genes significantly upregulated increased to 5,408, with 2,163 unique to this time point. Notably, 2,267 genes with significantly increased expression were shared across all three time points.

Conversely, IL10 macrophages displayed fewer transcriptional changes. At 6 h, 291 genes showed significantly increased expression, with 104 being unique. At 24 h, 344 genes were significantly upregulated (111 unique), with this number rising to 646 by 48 h, including 464 unique genes. Only 98 genes showed consistently significant upregulation across all three time points. In turn, macrophages polarized with conditioned medium of HepG2 (Sec-HepG2) showed minimal transcriptional changes early on, with 45 genes showing significant upregulation (18 unique) at 6 h, and 37 (11 unique) at 24 h. However, by 48 h, the number of genes with significant upregulation increased to 414, with 382 of these unique to this time point. Only 11 genes were consistently significantly upregulated across all three time points. In summary, IFN-γ_LPS polarization resulted in a markedly greater number of differentially expressed genes and more pronounced transcriptional changes than IL10 or Sec-HepG2 polarization, suggesting a more robust cellular response to IFN-γ_LPS treatment.

Many of the genes with significantly increased expression correspond to classical polarization markers for each stimulus. For each macrophage group, a gene set including the top 10 genes with FDR< 0.05 and the highest log_2_FC was selected. Macrophages treated with IFN-γ_LPS induced the expression of *CXCL9* and *CXCL10*, which are associated with inflammatory macrophages (**Figure 2B**) (33). Conversely, macrophages treated with IL10 overexpressed genes such as *CD163*, and Sec-HepG2 expressed significant genes such as *IL6* which are classical markers of anti-inflammatory macrophages (33).

To further explore the biological functions associated with the observed changes in gene expression, we performed GSEA on the differentially expressed gene list. **Figure 2C** illustrates the top five enriched pathways for each macrophage group at 24 h (normalized enriched score (NES)> 1 or NES< -1, adjusted *p* < 0.05). These pathways are all closely associated with immune responses and indicate functional activation among the three types of macrophages.

### 3.3 Genes selected by the CDBGE selector show improved performance in macrophage profiling compared with established methods

**Figure 3:**
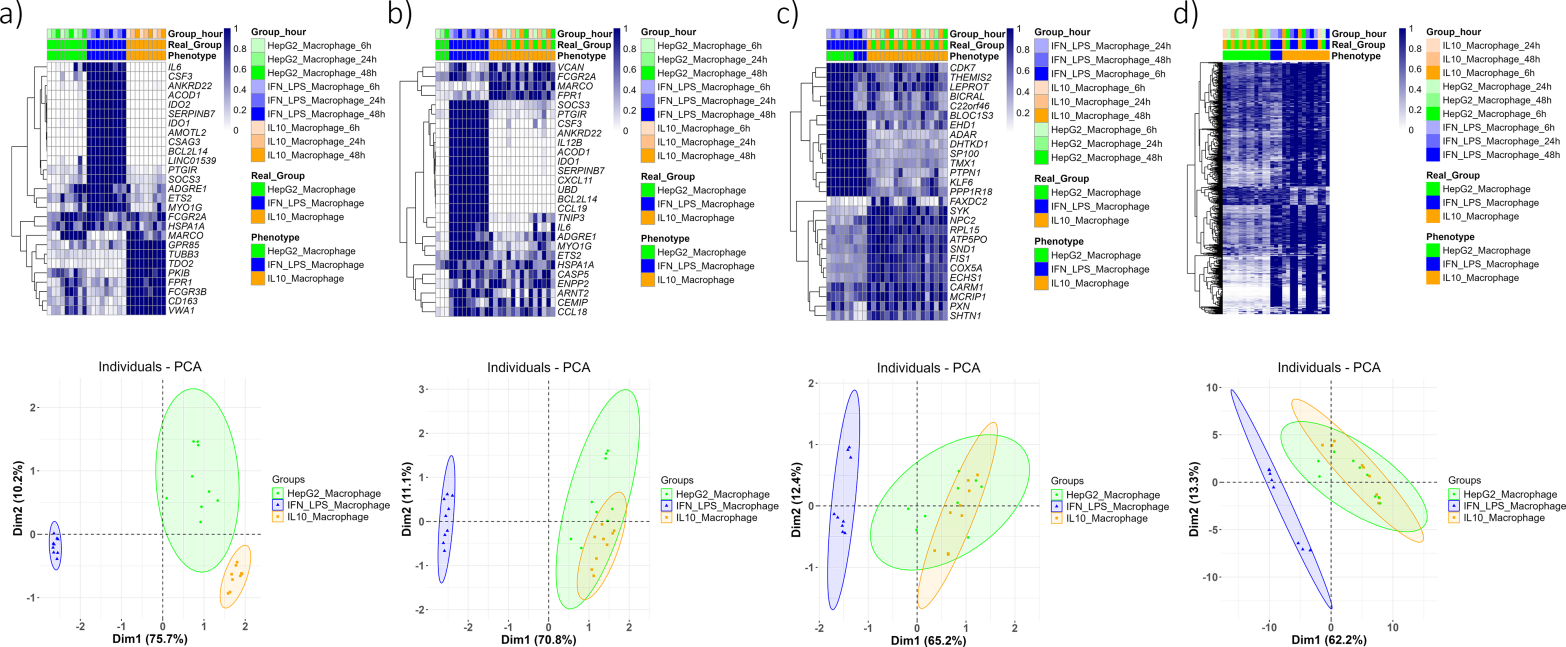
Comparison of markers selected by established methods and the CDBGE selector for their application in macrophage profiling. Top panel: Heatmaps of markers selected to characterize different types of macrophages using **(A)** CDBGE, **(B)** conventional, **(C)** Random Forest and **(D)** WGCNA methods. Performance is illustrated by comparing the true group of each sample (Real group, second row in heatmap) with the phenotype assigned based on the selected markers (third row in heatmap). Lower panel: PCA plots of macrophage profiling using the methods indicated above. Dim, dimension.

We next applied the CDBGE selector to our mRNA-seq data of human macrophage *in vitro* polarization. CDBGE selected an *n_g_* = 9 gene set for each condition (**Table 1**), hereafter referred to as the CDBGE gene set. This value was fixed to 9 due to constraints arising from the conventional method and to facilitate a direct comparison between both. Heatmap, PCA and confusion matrix analyses determined that the CDBGE gene set correctly assigned 100% all three macrophage types to their respective groups (**Figure 3A**, **Supplementary Table 1**).

**Table 1:** Gene sets identified by the conventional method (blue) and CDBGE selector (green), related to **Figure 2**.

Conventional method						
IFN-γ_LPS_Markers						
Time	6 h		24 h		48 h	
Gene	Log_2_FC	FDR	Log_2_FC	FDR	Log_2_FC	FDR
*ACOD1*	15.57	1.43·10^−20^	14.70	3.37·10^−81^	13.07	4.02·10^−93^
*IDO1*	14.10	1.03·10^−258^	13.80	2.66·10^−197^	13.80	3.58·10^−285^
*BCL2L14*	14.10	6.13·10^−09^	12.81	1.29·10^−22^	13.56	5.69·10^−28^
*CXCL11*	17.25	4.39·10^−21^	12.29	2.55·10^−68^	9.77	1.25·10^−10^
*IL12B*	17.22	1.26·10^−14^	12.76	3.23·10^−40^	8.96	9.43·10^−07^
*SERPINB7*	11.71	3.76·10^−02^	12.81	1.84·10^−22^	14.07	3.13·10^−12^
*CSF3*	13.70	1.27·10^−02^	12.45	8.89·10^−07^	12.03	7.59·10^−06^
*UBD*	12.78	3.41·10^−07^	11.61	3.65·10^−10^	12.56	1.98·10^−16^
*CCL19*	14.06	4.30·10^−39^	12.25	1.50·10^−61^	10.48	9.76·10^−107^
IL10_Markers						
Time	6 h		24 h		48 h	
Gene	Log_2_FC	FDR	Log_2_FC	FDR	Log_2_FC	FDR
*CEMIP*	6.41	2.19·10^−04^	7.23	1.51·10^−04^	5.24	1.62·10^−03^
*CASP5*	5.90	1.34·10^−13^	5.94	1.09·10^−10^	6.09	6.01·10^−14^
*TNIP3*	6.80	3.31·10^−56^	6.60	1.04·10^−04^	3.65	3.20·10^−04^
*ARNT2*	5.24	3.38·10^−18^	5.86	2.32·10^−18^	4.54	2.42·10^−11^
*ENPP2*	4.64	1.47·10^−57^	4.46	1.71·10^−20^	6.29	2.94·10^−150^
*VCAN*	4.38	2.70·10^−23^	3.89	5.63·10^−12^	4.10	1.90·10^−02^
*MARCO*	3.30	3.13·10^−02^	3.75	1.68·10^−03^	5.13	4.83·10^−96^
*CCL18*	3.70	1.06·10^−09^	3.72	9.41·10^−10^	4.50	1.99·10^−30^
*FPR1*	4.09	6.28·10^−36^	3.44	6.08·10^−40^	4.16	1.15·10^−42^
HepG2_Markers						
Time	6 h		24 h		48 h	
Gene	Log_2_FC	FDR	Log_2_FC	FDR	Log_2_FC	FDR
*IL6*	9.34	1.03·10^−09^	7.32	4.60·10^−11^	5.02	3.87·10^−09^
*ANKRD22*	3.79	1.95·10^−02^	3.07	2.74·10^−02^	3.79	2.68·10^−09^
*ADGRE1*	2.45	2.01·10^−02^	2.54	7.65·10^−06^	3.28	8.83·10^−16^
*SOCS3*	3.32	5.56·10^−03^	2.55	8.60·10^−06^	2.31	1.44·10^−03^
*MYO1G*	2.61	1.05·10^−02^	2.06	4.14·10^−02^	1.64	4.77·10^−03^
*PTGIR*	2.34	2.53·10^−02^	2.15	2.31·10^−03^	1.78	4.29·10^−03^
*FCGR2A*	1.96	7.40·10^−03^	1.88	9.71·10^−03^	2.28	4.49·10^−06^
*ETS2*	2.36	7.40·10^−03^	1.89	6.41·10^−04^	1.68	5.46·10^−03^
*HSPA1A*	1.78	1.67·10^−02^	1.39	2.74·10^−02^	1.06	3.21·10^−03^
CDBGE						
IFN-γ_LPS_Markers						
Time	6 h		24 h		48 h	
Gene	Log_2_FC	FDR	Log_2_FC	FDR	Log_2_FC	FDR
*CSAG3*	7.24	6.44·10^−03^	7.27	1.68·10^−04^	7.82	8.23·10^−07^
*CSF3*	13.70	1.27·10^−02^	12.45	8.89·10^−07^	12.03	7.59·10^−06^
*LINC01539*	7.12	7.86·10^−03^	7.48	9.67·10^−05^	10.10	2.64·10^−14^
*SERPINB7*	11.71	3.76·10^−02^	12.81	1.84·10^−22^	14.07	3.13·10^−12^
*BCL2L14*	14.10	6.13·10^−09^	12.81	1.29·10^−22^	13.56	5.69·10^−28^
*IDO1*	14.10	1.03·10^−258^	13.80	2.66·10^−197^	13.80	3.58·10^−285^
*AMOTL2*	12.80	2.13·10^−02^	10.40	5.43·10^−14^	8.84	4.85·10^−10^
*ACOD1*	15.57	1.43·10^−20^	14.70	3.37·10^−81^	13.07	4.02·10^−93^
*IDO2*	1.65	2.24·10^−06^	10.29	2.01·10^−14^	11.24	7.89·10^−19^
IL10_Markers						
Time	6 h		24 h		48 h	
Gene	Log_2_FC	FDR	Log_2_FC	FDR	Log_2_FC	FDR
*TUBB3*	3.28	1.12·10^−14^	3.46	1.47·10^−36^	2.68	1.15·10^−10^
*TDO2*	3.70	2.60·10^−06^	4.23	1.16·10^−08^	2.80	2.38·10^−05^
*CD163*	2.09	5.00·10^−10^	2.48	1.22·10^−10^	2.92	6.17·10^−29^
*VWA1*	3.23	1.88·10^−08^	2.58	3.55·10^−04^	2.41	2.59·10^−04^
*GPR85*	3.66	2.24·10^−04^	3.24	7.13·10^−04^	2.81	1.28·10^−2^
*FPR1*	4.09	6.28·10^−36^	3.44	6.08·10^−40^	4.16	1.15·10^−42^
*MARCO*	3.30	3.13·10^−02^	3.75	1.68·10^−03^	5.13	4.83·10^−96^
*PKIB*	2.21	6.25·10^−04^	2.43	2.79·10^−06^	2.52	8.18·10^−09^
*FCGR3B*	1.88	2.68·10^−06^	2.32	1.06·10^−07^	2.58	1.87·10^−18^
HepG2_Markers						
Time	6 h		24 h		48 h	
Gene	Log_2_FC	FDR	Log_2_FC	FDR	Log_2_FC	FDR
*IL6*	9.34	1.03·10^−09^	7.32	4.60·10^−11^	5.02	3.87·10^−09^
*ANKRD22*	3.79	1.95·10^−02^	3.07	2.74·10^−02^	3.79	2.68·10^−09^
*ADGRE1*	2.45	2.01·10^−02^	2.54	7.65·10^−06^	3.28	8.83·10^−16^
*SOCS3*	3.32	5.56·10^−03^	2.55	8.60·10^−03^	2.31	1.44·10^−03^
*MYO1G*	2.61	1.05·10^−02^	2.06	4.14·10^−02^	1.64	4.77·10^−03^
*PTGIR*	2.34	2.53·10^−02^	2.15	2.21·10^−03^	1.78	4.29·10^−03^
*FCGR2A*	1.96	7.40·10^−03^	1.88	9.71·10^−03^	2.28	4.49·10^−06^
*ETS2*	2.36	7.40·10^−03^	1.89	6.41·10^−04^	1.68	5.46·10^−03^
*HSPA1A*	1.78	1.67·10^−02^	1.39	2.74·10^−02^	1.06	3.21·10^−03^

Log_2_FC and FDR values are shown for different cell types at various time points.

To benchmark the performance of the selector, we compared it against a conventional 9 gene set derived from the top differentially expressed genes with the highest log_2_FC and an FDR< 0.05, through heatmap analyses (**Table 1**, **Figure 3B**). The value for *n_g_* was limited by the pre-selection of HepG2 genes, since only 9 genes satisfied both log_2_FC>1 and FDR< 0.05 criteria. This tool classified 78% of macrophage types (IFN-γ_LPS, IL10 and Sec-HepG2). While it accurately identified IFN-γ_LPS macrophages, it showed poor discrimination between IL10 and Sec-HepG2 macrophages, resulting in frequent misclassification. Likewise, using the gene set selected by Random Forest and WGCNA analysis, only 44% of macrophage groups were correctly assigned (**Supplementary Table 1**). These differences in performance were further supported by PCA: Random Forest and WGCNA similarly showed overlap between IL10 and Sec-HepG2 macrophages, while IFN-γ_LPS macrophages clustered closely with these groups, further indicating poor separation (**Figures 3C, D**, respectively).

### 3.4 The CDBGE algorithm enables analysis of macrophage polarization dynamics

**Figure 4:**
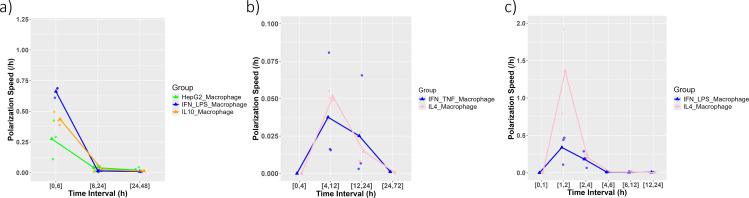
Macrophage polarization dynamics analysis using the CDBGE selector. Speed plots of macrophage polarization changes over time of: **(A)** our data, GSE331143; **(B)** the human macrophage GSE16385 database; and **(C)** the murine macrophage GSE158094 database.

The CDBGE also enabled analysis of changes in macrophage gene expression over time, thereby providing valuable insights into polarization activity. **Figure 4** illustrates macrophage polarization speed across different experimental datasets. Using our own data, during the initial 24 h, a sharp decline in the rate of change was observed across all macrophage phenotypes, indicating an initial period of rapid cellular response (**Figure 4A**).

Consistent results were obtained when this analysis was applied to the publicly available human macrophage dataset (GSE16385), which included time points ranging from 0 to 72 h (30). As in our *in vitro* experiment, the first 24 h showed substantial changes in dynamics, suggesting that macrophage polarization *in vitro* is most pronounced during the initial 24 h, since after 24 h the speed reaches 0 (**Figure 4B**). However, the same analysis applied to *in vitro* polarized murine macrophages (GSE158094) (29) revealed a slightly different dynamic profile, in which these cells showed a faster early polarization rate than human macrophages (**Figure 4C**). In that case, a significant rate of change was observed within the first 6 h, after which this speed stabilized and approached zero, indicating a shift towards a more stable macrophage polarization state.

### 3.5 Confirmation of the accurate and flexible performance of the CDBGE selector on independent data

**Figure 5:**
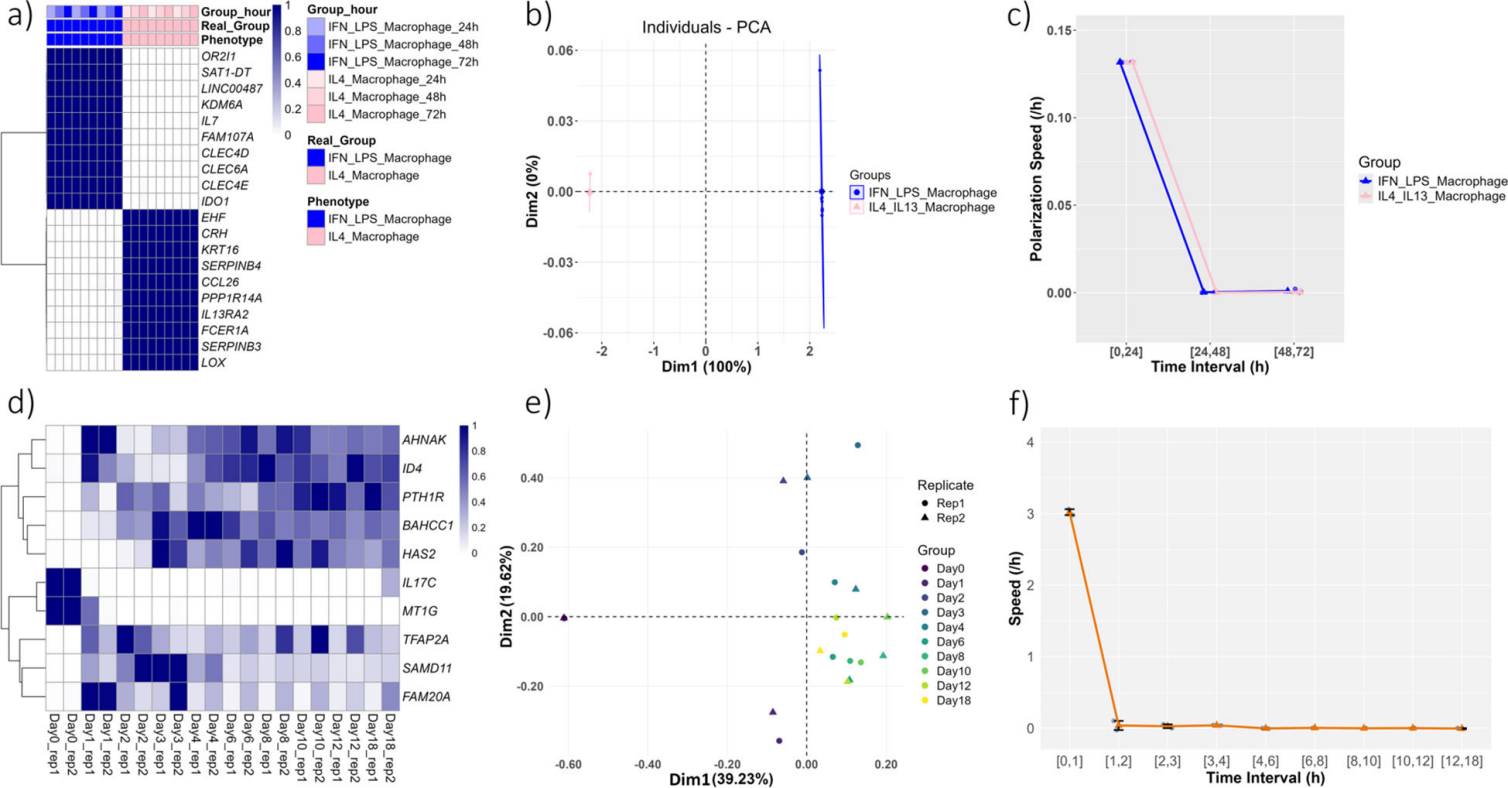
Broad applicability of the CDBGE selector across public datasets. Application of the CDBGE selector to the human macrophage GSE273627 dataset (upper row) and the hESC differentiation GSE274620 dataset (lower row). **(A, D)**, Heatmaps of genes selected using the CDBGE selector. **(B, E)**, PCA plots showing classification of cell groups based on selected markers. **(C, F)**, speed plot of changes in gene dynamics.

We used the CDBGE selector on gene data from two additional independent public datasets (Migliaccio et al., GSE273627) (31) and (Keskin et al., GSE274620) (32), using a *n_g_* = 10 gene set for each condition. According to the heatmap analysis, the CDBGE successfully distinguished among the macrophage phenotypes from the study by Migliaccio et al. (31) (**Figure 5A**). PCA (**Figure 5B**) and confusion matrix (**Supplementary Table 1**) confirmed that the phenotypes were clearly separated, supporting the robustness of the selector. Additionally, analysis of dynamics confirmed the importance of the early 24 h time window, during which the most significant changes in macrophage polarization occurred (**Figure 5C**).

In the study by Keskin et al. (GSE274620) (32), CDBGE was used to select 10 key genes to monitor hESC differentiation dynamics. Heatmap analysis revealed distinct gene expression patterns across time points. At day 0, only two genes, IL17C and MT1G, were highly expressed. By day 1, the number of expressed genes increased, displaying a profile distinct from that observed at days 2-3, which exhibited similar expression patterns. From day 4 to day 18, expression patterns stabilized (**Figure 5D**). PCA positioned day 0 cells along the primary axis, whereas day 1 cells formed a distinct cluster in a separate quadrant. Cells from days 2–3 clustered closely together, and those from days 4–18 occupied a nearby, more compact region, reflecting coordinated transcriptional programs during differentiation (**Figure 5E**). Speed analysis further highlighted dynamic changes over time. The transition from day 0 to day 1 was associated with rapid transcriptional shifts, whereas changes between days 2–4 were more gradual. From day 6 onward, transcriptional dynamics appeared to stabilize, consistent with sustained differentiation (**Figure 5F**). These results demonstrate that the method captures both temporal progression and stage-specific dynamics during hESC differentiation (32).

## 4 Discussion

The heterogeneity of macrophages and the critical involvement of these cells in shaping disease prognosis underscore the necessity for an in-depth characterization of their transcriptional landscapes. Cancer exemplifies this complexity, wherein the dynamic cues of the TME orchestrate the pro-tumorigenic phenotypes and functions of macrophages. In this context, we proposed that a selector based on gene expression could provide valuable insights into macrophage heterogeneity and thereby advance our understanding of the roles of these cells in disease progression. Our results highlight the power of integrating advanced mathematical modeling with gene expression analysis to resolve macrophage heterogeneity and temporal dynamics. By applying a Cartesian distance-based framework, we not only successfully identified gene sets that characterize distinct macrophage types with precision but also captured the trajectory of gene expression over time, identifying critical transition points during polarization. This approach transforms static snapshots of gene expression into a dynamic, multidimensional view of cellular states, providing insights that are inaccessible with conventional methods.

To develop the CDBGE selector, we used two independent datasets generated with *in vitro* stimulated macrophages from different species – human and murine, each involving distinct stimuli and varying numbers of replicates. Despite these differences, the CDBGE selector consistently showed a robust performance, achieving a higher proportion of correctly classified samples across the groups compared with other established methods, as confirmed by PCA analysis and confusion matrices. This result indicated that the CDBGE selector was efficiently built to identify the underlying patterns and that its parameters and design were appropriate. Additionally, this robustness suggests that the method is largely independent of replicate number or species, thereby highlighting its broad applicability and reliability across diverse experimental conditions. However, one limitation of this CDBGE selector is that it requires the definition of a reference group for comparison analysis, like the M-CSF that were used in this study.

The success of this approach relies not only on its performance as gene selector but also on the simplicity, interpretability, and versatility of the model. The Cartesian distance between two points is an understandable concept that can be readily extended to an n-dimensional space, also allowing for the definition of an average change in speed as the ratio between distance and time. The normalization that precedes the proposed distance calculation enables the detection of specific genes that, although may not exhibit a large absolute 10-fold change, are representative of significant changes induced by the stimuli. The Jensen–Shannon distance, a symmetrized and smoothed variant of the Kullback-Leibler divergence, is based on changes in entropy (34). Although this method was also evaluated in preliminary studies, its performance was inferior, with only 83% of groups correctly classified, compared with CDBGE selector.

To validate our model, a new database was used to evaluate its performance. To this end, we utilized a dataset generated *de novo*, which included human macrophages stimulated *in vitro* with 1 pro-inflammatory (IFN-γ_LPS) and 2 immunosupressive stimuli, over time. Since this dataset was created in-house, the experimental conditions were tightly controlled and well characterized. DGE analysis of these samples revealed a substantial number of significantly regulated genes among the different macrophage groups. Notably, the number of significant genes increased when macrophages were exposed to cytokines for 48 h compared to 24 h and 6 h, demonstrating that the transcriptional response to stimuli is highly time-dependent. This identifies a critical window during which the stimulus exerts maximal effect and highlights the importance of selecting appropriate time points for studying macrophage populations *in vitro*. The observation that different genes exhibit distinct dynamics and peak expression at varying intervals underscores the value of high-resolution temporal profiling. More broadly, these results emphasize that capturing the full complexity of cellular responses requires methods that integrate both the magnitude and timing of gene expression changes (35).

Among the different conditions tested, macrophages stimulated with IFN-γ_LPS showed the highest number of differentially expressed genes (DEG) while Sec-HepG2 macrophages exhibited fewer significant changes. This result is consistent with previous studies, suggesting that the HepG2 secretome is a weaker stimulus than IFN-γ_LPS, or IL10 at the concentrations used (12). The secretome is a complex mixture containing hundreds of molecules, including proteins, lipids, nucleic acids, metabolites, and other small molecules (36). In contrast, cytokines exert more direct and specific effects on immune cells, leading to a more pronounced and predictable response.

Importantly, well-established markers for macrophage polarization were among the top differentially expressed genes in each condition. IFN-γ_LPS macrophages showed strong upregulation of *CXCL9*, and *CXCL10*, which are pro-inflammatory markers (35). Conversely, IL10-treated macrophages showed increased expression of *CD163*, consistent with immunosupressive polarization (35) and Sec-HepG2 macrophages also showed upregulation of immune-related genes, including *IL6*. The additional GSEA confirmed the biological relevance of these responses, supporting the robustness of the *in vitro* model. Collectively, these findings highlight the distinct transcriptional and functional profiles among different macrophage activation states, confirming the reliability of our experimental system for subsequent analyses. Interestingly, among the CDBGE gene sets, 15 genes with not evident roles on macrophage polarization state classification were identified, namely *CSAG3, LINC01539, SERPINB7, BCL2L14, AMOTL2, TUBB3, TDO2, VWA1, GPR85, PKIB, FCGR3B, HSPA1A, ADGRE1, MYO1G* and *PTGIR*. These findings show that the CDBGE selector not only highlighted the most characteristic genes defining distinct macrophage types but also uncovered these 15 new candidate genes, which play key roles in macrophage identity and function.

Moreover, our data show that, using the new CDBGE method, we achieved an improvement in the selection of gene sets that characterize each polarization state, as shown by a higher performance of subsequent macrophage classification when compared to other well-established methods like the conventional based on fold-change, Random Forest and WGCNA methods, increasing the proportion of correctly classified samples from 78% and 44% to 100%. In particular, CDBGE is capable of eliminating misclassification between the IL10 and Sec-HepG2 gene sets. This method integrates the distance concept, enabling simultaneous analysis of multiple cell groups by representing them as points in a multidimensional space. This mathematical formulation allows direct comparison among diverse polarization conditions, yielding a more coherent and biologically meaningful classification. By moving beyond pairwise contrasts, the CDBGE selector provides a powerful framework for uncovering subtle transcriptional relationships that escape the other established approaches we have analyzed.

We acknowledge that the small sample size (N = 3 per condition) in our in-house experiment represents a statistical limitation regarding the estimation of uncertainty and out-of-sample validation. However, this framework mirrors common experimental conditions in immunology, where human donor samples are subject to inherent logistical constraints. To mitigate this, we prioritized cross-dataset validation rather than relying on a single cohort. The fact that the CDBGE selector maintained its performance across multiple independent datasets and species reinforces the robustness of the method for identifying gene panels and demonstrates its reliability beyond the initial training data.

The algorithm produced different gene lists when applied to different human macrophage databases. This variation likely reflects differences in experimental design, including the type and concentration of stimuli used to activate macrophages. Such specificity can influence the expression profiles captured by the CDBGE selector, particularly given the high plasticity of these cells and their sensitivity to cytokine dosage and treatment conditions. These findings underscore the importance of experimental standardization and context-aware interpretation when comparing datasets derived from heterogeneous stimulation protocols.

Another limitation of our approach is its dependence on the initial differential-expression filtering, because the number of genes available for macrophage cluster classification is determined by this pre-selection step. After applying the filtering criteria, few genes of Sec-HepG2 remained, and these genes were therefore used for macrophage classification. To examine the robustness of this choice, we compared different thresholds and relaxed the pre-selection to include genes that were significant at 48 h. Polarization dynamic analysis have suggested that 48 h represents a stage at which macrophage polarization begins to stabilize, which may explain why a larger number of DEGs are detected at this time.

To evaluate the effect of gene number, we tested multiple gene sets (n_g_=5, 10, 15, 20, 50) for macrophage pattern identification using both methods. Across all scenarios, CDBGE consistently yielded superior performance (**Supplementary Figure S1**). These results indicate that CDBGE is highly versatile and can adapt to different DGE conditions, whether based on stringent criteria across all time points or on significance at a single time point. Moreover, the method performs well across different gene set sizes, suggesting that the optimal number of genes should be chosen according to the specific objective of the study.

A key advantage of the CDBGE selector is its capacity to analyze the rate at which macrophages exposed to different stimuli evolve into distinct phenotypes. In this study, we successfully applied it to study the dynamics of macrophage polarization into multidimensional aspect, considering each stimuli gene cluster as a separate dimension. This multidimensional view provides a broader perspective for understanding polarization activities, and it allows the subsequent estimation of polarization speed to be more accurate and reflect greater complexity. In human macrophages, polarization activity was significant during the initial 24 h, as indicated by a notable change in its speed. Subsequently, after 24 h, the polarization speed approaches zero. This finding aligns with previous proteomics analysis of the first 24 h of treatment with IL4 or IFN-γ_LPS as the phase of polarization induction (37). In contrast, murine macrophages showed polarization activity earlier, within the first 6 h, suggesting that cellular production should be studied specifically during this period (38). These results highlight that macrophage polarization dynamics may be species-specific. However, gene expression dynamics studies from additional murine macrophage experiments are needed to confirm this finding. It should be noted that the normalization protocol applied to the data, which is specific to each time point, amplifies the early polarization dynamics and could bias the speed evaluation. Nevertheless, using a global normalization procedure that considers all time points when identifying the maximum value yields similar speed profiles, thereby reinforcing the conclusions of this study.

Our study also showed that the CDBGE selected genes not only improved macrophage classification based on DGE but also generalized across datasets and cell types, providing a powerful alternative framework for DGE analysis. In this regard, the application of the CDBGE selector to a stem cell differentiation study *in vitro* (32) revealed that certain time points exhibited similar transcriptional profiles, whereas others were clearly separated, reflecting distinct differentiation states. In contrast to the conventional method, the CDBGE selector facilitates the study of differentiation dynamics by tracking gene expression changes over time, thereby providing a better understanding of the temporal aspects of differentiation, which is a key feature of stem cell development. By identifying informative gene markers, this approach may also advance the characterization of cellular states and provide a new tool for clinical studies comparing distinct patient cohorts.

To conclude, we developed a new method to distinguish cellular expression dynamics on the basis of DGE analysis, significantly improving the accuracy of other established approaches. The CDBGE selector provides a robust and versatile framework for DGE analysis, enabling the identification of both established and novel markers. By integrating multidimensional and temporal information, this approach extends beyond conventional differential expression methods, offering deeper insights into cellular heterogeneity and dynamic biological processes. Finally, the application of the CDBGE selector across multiple datasets produced consistently strong results, demonstrating its broad applicability and potential as a versatile framework for gene expression and cell-type analysis.

## Data Availability

The data supporting the findings of this study are available from the corresponding author upon reasonable request. mRNAseq data have been deposited in Gene Expression Omnibus (GEO) database and will be accessible through GEO accession GSE331143. The code used in this study is available in a public GitHub repository (https://www.ncbi.nlm.nih.gov/geo/query/acc.cgi?acc=GSE331143).
