# Supplementary material for: A versatile distance-based approach for gene expression selection across diverse biological systems
Source: Front Immunol. 2026 Jul 13;17:1843796. doi: 10.3389/fimmu.2026.1843796 (PMC13402154; doi:10.3389/fimmu.2026.1843796)
Supplement: Supplementary file 1 [file DataSheet1.pdf]

Supplementary Material

1 Supplementary Figure 1

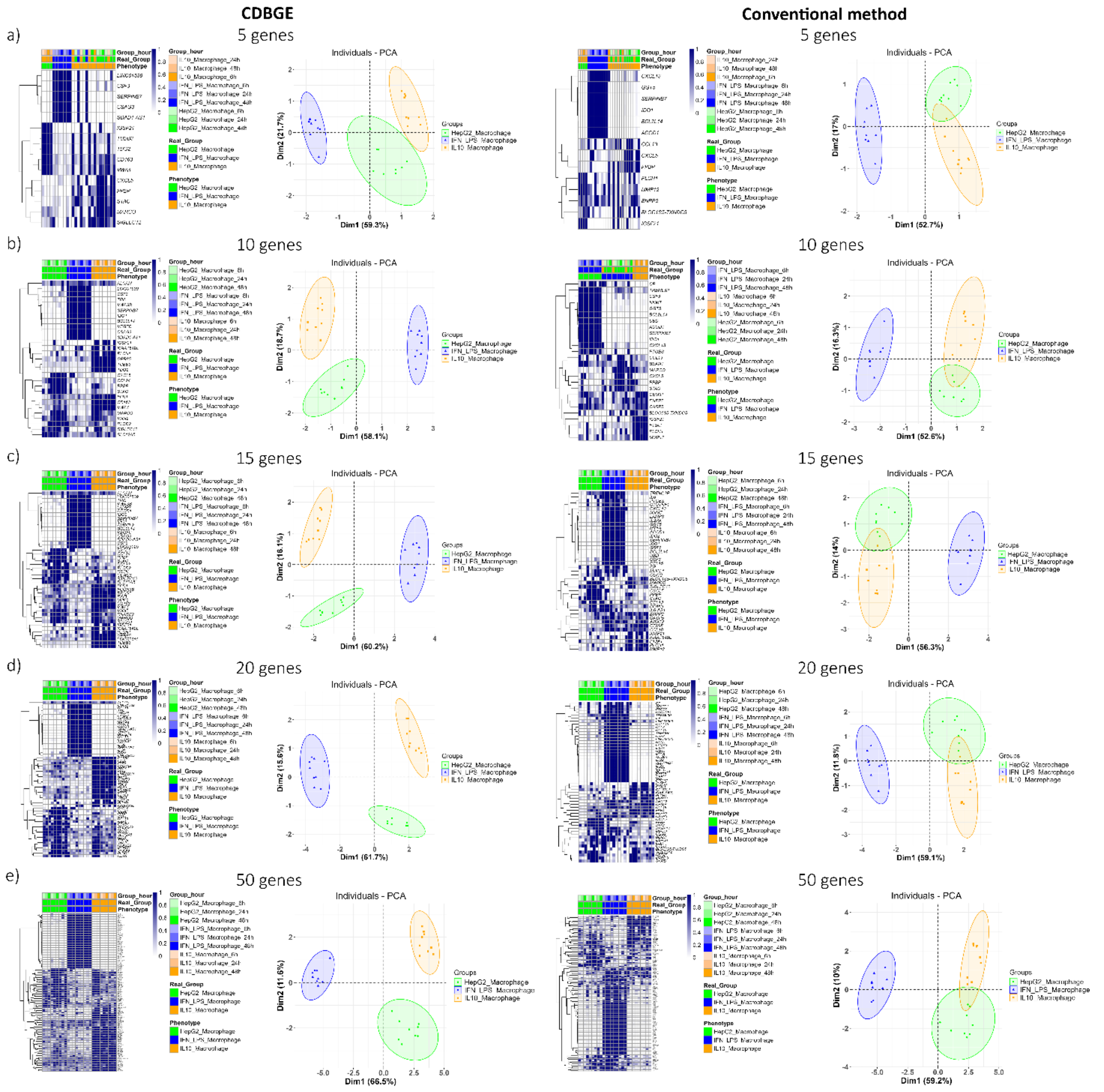

Supplementary Figure 1. Selection using multiple gene-set numbers for macrophage profiling. Heatmaps and PCA plots for the n= 5, 10, 15, 20 and 50 gene-sets. Results obtained using CDBGE selector are shown on the left, and those using the conventional method on the right. In PCA, percentages represent variance captured by Dimension (Dim) 1 and Dim 2.
