# Supplementary figures and images for: A versatile distance-based approach for gene expression selection across diverse biological systems
Source: Front Immunol. 2026 Jul 13;17:1843796. doi: 10.3389/fimmu.2026.1843796 (PMC13402154; doi:10.3389/fimmu.2026.1843796)

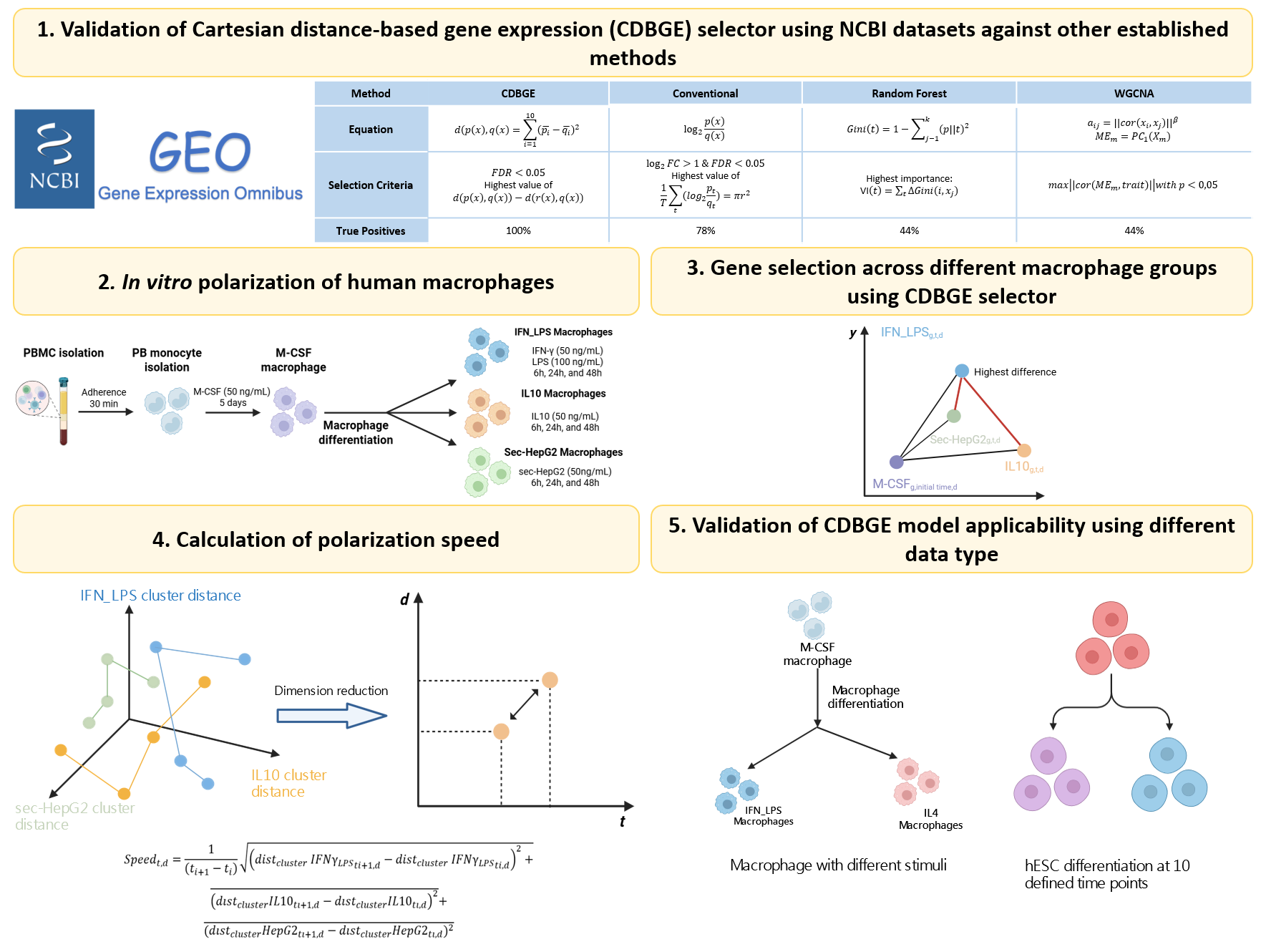

Supplement: Supplementary file 2 [file Image1.tif]
